# A recombinase polymerase amplification lateral flow assay (RPA-LF) for detection of *Babesia* *divergens-*like parasites

**DOI:** 10.1186/s13071-026-07549-0

**Published:** 2026-07-27

**Authors:** Ernest Chin Jia Bin, Keir Hughes, Cheryl Whitehorn, Mia White, Kayleigh Hansford, Jolyon Medlock, Ellen Knuepfer, Taane G. Clark, Mojca Kristan, Susana Campino

**Affiliations:** 1 Faculty of Infectious and Tropical Diseases, London School of Hygiene and Tropical Medicine (LSHTM), Keppel Street, London, WC1E 7HT UK; 2 Teaching and Diagnostic Unit, Faculty of Infectious and Tropical Diseases, London School of Hygiene and Tropical Medicine (LSHTM), Keppel Street, London, WC1E 7HT UK; 3 Diagnostics & Pathogen Characterisation, UKHSA, Porton Down, Salisbury, SP4 0JG UK; 4 Medical Entomology and Zoonoses Ecology, UKHSA, Porton Down, Salisbury, SP4 0JG UK; 5 The Royal Veterinary College, Hawkshead Lane, Hatfield, AL9 7TA UK

**Keywords:** *Babesia divergens*, RPA-LF, Recombinase polymerase amplification, Babesiosis, Lateral flow, Urban Green space

## Abstract

**Background:**

*Babesia divergens* is a tick-borne apicomplexan parasite found predominantly in Europe, causing babesiosis in livestock and occasionally in humans, with severe disease reported in immunosuppressed or asplenic individuals. As populations of *Ixodes ricinus*, the principal vector, expand geographically, including into urban environments, there is a growing need for rapid, sensitive, and field-deployable surveillance tools. In this study, we aim to developed a Recombinase Polymerase Amplification Lateral Flow (RPA-LF) assay for the detection of *B. divergens*.

**Methods:**

RPA assays targeting *B. divergens hsp70*, *cox1*, and *18S rRNA* genes were designed and evaluated for sensitivity and specificity using serial dilutions and control samples, including other apicomplexan parasites and tick samples spiked with *B. divergens* DNA. To assess field performance, 279 *I. ricinus* ticks were collected from urban parks in Greater London and screened by qPCR and a subset also tested using the new RPA-LF assays. *Anaplasma phagocytophilum* was screened by qPCR but not detected.

**Results:**

The *cox1* and *18S rRNA* RPA-LF assays demonstrated high specificity, with detection limits of at least 10^−5^ ng/μl and 10^−3^ ng/μl, respectively, under isothermal conditions (39 °C for 30 min). No cross-reactivity was observed with non-target DNA. Field validation using pooled samples revealed a single sample positive for *Babesia divergens* by qPCR, indicating an estimated nymphal infection prevalence of 0.4–1.2%, as previously observed in UK tick studies. The *18S rRNA* RPA-LF assay successfully detected this pool, whereas the *cox1* RPA-LF did not. Sanger sequencing confirmed the presence of *B. divergens*/*B. capreoli*-like DNA, although species-level discrimination was limited due to high sequence similarity.

**Conclusions:**

We report the first successful development of an RPA-LF assay for the detection in ticks of *B. divergens* or the closely related species *B. capreoli*. The assay demonstrates high sensitivity and specificity under isothermal conditions and provides a portable and reliable tool for field-based molecular surveillance of emerging tick-borne pathogens. Field validation revealed *B. divergens*/*B. capreoli* in questing ticks from urban green spaces in London, underscoring the importance of this tool for early detection and ongoing surveillance of tick-borne parasites in expanding urban ecosystems.

**Graphical Abstract:**

**Figure:**
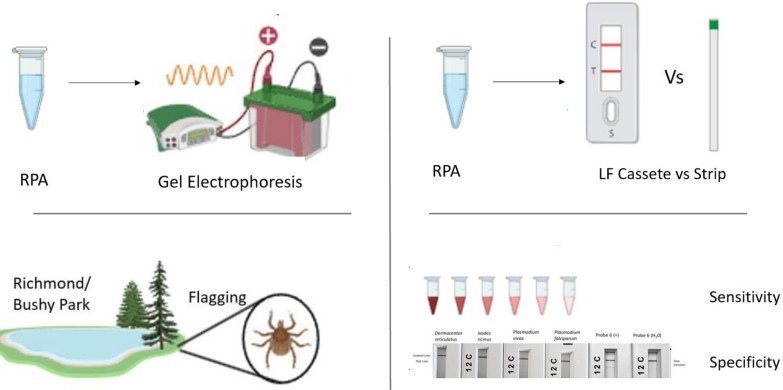

**Supplementary Information:**

The online version contains supplementary material available at 10.1186/s13071-026-07549-0.

## Background

Transmitting a wider variety of pathogens than any other arthropod vector, ticks are second only to mosquitoes in vectoring human and veterinary-relevant pathogens[1]. Ticks can transmit parasites, such as *Babesia divergens* (bovine and human babesiosis), bacteria, such as *Borrelia burgdorferi* sensu lato (Lyme disease) and *Anaplasma phagocytophilum* (human granulocytic anaplasmosis and pasture fever), and viral pathogens, such as tick-borne encephalitis virus (TBEV). In addition, tick bites can trigger severe allergic reactions, such as alpha-gal syndrome [2].

In recent decades, expanding ranges of ticks [3], environmental changes [4], and the increasing prevalence of coinfection with multiple pathogens within tick populations [5] have fuelled rises in both the distribution and density of tick-borne diseases (TBD) [6], raising significant public health concerns and fuelling the initiation of various tick surveillance schemes. Despite this growing threat of TBDs in the face of increased tick/human contact, European surveillance efforts largely focus on screening for *Borrelia burgdorferi,* as Lyme disease is the most reported and economically significant TBD in the northern hemisphere [7–9]. These efforts have inevitably overshadowed and overlooked other clinically important pathogens, such as *Babesia divergens*.[10]

Historically associated with bovine babesiosis throughout cattle-grazing areas of Europe, *Babesia* parasites are intraerythrocytic piroplasms that are now recognised as severe human pathogens, potentially life-threatening in immunocompromised individuals [11, 12]. While only 60 confirmed cases of human babesiosis have been reported in Europe, and in UK a recent case in Devon [10, 13], the asymptomatic seropositivity in patients suggests significant underdiagnosis and under-reporting [10, 11]. This is particularly important when considering the potential severity in patients with *Borrelia burgdorferi* coinfections [5] as high tick exposure individuals are more likely seropositive for multiple TBDs simultaneously [5]. Once established in tick vectors, *Babesia* parasites can persist through transstadial and transovarial transmission, allowing infected ticks to maintain the pathogen without requiring reinfection from vertebrate hosts during their development, thereby enhancing transmission potential [11]. In the UK and Europe, *B. divergens* transmission is primarily facilitated by *Ixodes ricinus*. Capable of transmitting *B. microti, B. venatorum* and *B. capreoli*[10, 14], its extensive range spans from Scandinavia to North Africa [2, 5], making it the most widespread and epidemiologically significant tick species in Europe for babesiosis transmission. Furthermore, localised outbreaks of autochthonous canine babesiosis in Essex, South Wales and Devon [14, 15], transmitted by *Dermacentor reticulatus*, underline the need for molecular xenosurveilance, the detection of pathogen DNA/RNA in vectors, to monitor emerging TBDs.

Existing systems used to screen ticks for pathogens are primarily PCR-based, which can be inaccessible without specialist infrastructure and technical expertise [2, 16]. While such systems have their place as part of established surveillance programmes, the development of a rapid, low-resource, field-deployable molecular assay would provide an additional tool for current surveillance efforts [9, 17, 18]. Such complementary diagnostic approaches would enhance surveillance capabilities by enabling preliminary on-site screening, particularly in resource-limited settings or remote locations, where conventional PCR workflows are not feasible while maintaining the option for confirmatory testing using established laboratory-based methods.

Recombinase polymerase amplification (RPA) presents an alternative to PCR for application in field settings, as it requires minimal technical investment due to its rapid DNA amplification (30 min) at constant low temperatures (~ 39 °C), thereby eliminating the need for expensive thermocyclers [19]. Other isothermal methods pose their own unique obstacles, such as loop-mediated isothermal amplification (LAMP), which requires high temperatures (65˚C), complex primer design, and frequent, nonspecific amplification [16], while helicase-dependent amplification (HDA) requires complex buffer optimisation [20]. Thus, RPA is the most promising point-of-need, isothermal molecular assay suited for rapid pathogen surveillance as it operates quickly at a temperature range convenient for field situations [16, 20]. Field-portable RPA suitcases, containing the laboratory reagents and equipment needed, are also available to support the deployment of RPA at the point-of-need, expanding its capabilities to include resource-poor areas[16].

RPA has previously been used to detect *Babesia* in agriculture, human and veterinary medicine [19], including *Babesia vogeli, B. gibsoni, B. canis* [21, 22], *B. orientalis* [23] and *B. microti* [24]. Coupling RPA with lateral flow (LF) detection systems further enhances its utility as LF strips allow for rapid, equipment-free result interpretation, with gold nanoparticle-based probes providing signal amplification. In fact, LF-RPA assays targeting *B. vogeli* and *B. gibsoni* have demonstrated up to 10^5^-fold greater sensitivity than RPA products visualised via gel electrophoresis[21]. However, to date, no RPA assay has been developed for the detection of *Babesia divergens*.

Given the expansion of tick populations and the importance of *Babesia divergens* as a zoonotic pathogen, alongside the continued absence of field-portable molecular diagnostic tools, rapid isothermal detection assays would be critical for improving *B. divergens* surveillance methods. In this study, we aim to established a novel *Babesia divergens* RPA-LF assay offering a resource-limited alternative to PCR, specifically in the role of xenomonintoring using the presence of pathogen-positive ticks as a proxy for human/animal infection. Previous studies have detected *Babesia*-positive ticks via PCR in parts of Scotland, Wales, and England, with reported prevalence ranging from 0.2% to 0.38% (2014–2020) [10, 25, 26]. While *Babesia divergens* is primarily associated with cattle, this and other babesia species, have also been detected in deer popularions across Europe, including the UK [27, 28]. This suggests that deer-associated environments may act as overlooked reservoirs and contribute to the maintenance of *Babesia* transmission cycles.

As a proof-of-concept to validate the assay under field conditions, we screened ticks collected from two urban green spaces across Greater London, owing to their substantial deer population, past records of ticks, ease of public access and diversity of potential tick habitats, making them representative settings, where frequent human–tick interactions may elevate public health risk [1]. These results demonstrate the assay's potential application in routine surveillance and public health monitoring.

## Methods

### Tick collection and morphological identification

A total of 279 ticks (9 *Ixodes ricinus* adults, 249 *Ixodes ricinus* nymphs and 21 *Ixodes* nymphs) were successfully collected in June 2024 during the first seasonal peak in tick activity in summer [6, 9, 10, 27]. Considering the recognised risk of urban green spaces as known tick habitats in the UK [27, 28], Bushy Park and Richmond Park were selected (see Supplemental Information S1 for coordinates), as these parks have a substantial deer population, past records of ticks and are easy to access [1]. Questing ticks were collected using the flagging sampling method [29]. Nymphs were immediately transferred to tubes containing 70% ethanol [29]. Collected ticks were identified up to the life stage and species level using the identification keys, using a Leitz Laborlux S binocular microscope [3, 30, 31]. Ticks whose species could not be confidently determined were excluded from further study. Due to the challenges associated with identifying tick larvae [3] 31 and time constraints, the larvae were discarded.

### DNA extraction and quantification

DNA was extracted from ticks using the Qiagen DNeasy Blood and Tissue Kit (Qiagen, Germany). After storage, transport and identification, field samples were transferred to 100 µl of Milli-Q Water (Millipore Milli-Q system) in pools of 3 *Ixodes ricinus* nymphs. Small pool sizes maximised accurate minimum infection rates, pool prevalence and positivity estimates while balancing prevalence underestimation against pool dilution effects [32]. A total of 200 µl of ATL buffer was added, and samples were homogenised via crushing with pipette tips. Proteinase K (20 µl) was added, and each tube was incubated overnight at 56 °C to maximise lysis [33]. The subsequent steps were followed as per the manufacturer's instructions, except the elution of DNA was performed by adding 30 µl of buffer EB to concentrate DNA. After extraction, DNA was quantified using a Qubit DS-11 FX + Spectrophotometer/fluorometer (Denovix, USA) using the HS assay. DNA was also extracted from *B. divergens* (Rouen 1987) grown in vitro in human red blood cells (RBCs). RBCs were supplied by the NHS Blood and Transplant service as research red cells (approval by the research ethics committee, NHS HRA 21/YH/0085 and RVC URN 2021 20044-3). *B. divergens* infected RBCs at ~ 15% parasitaemia were lysed using 0.075% (w/v) saponin in PBS at room temperature to release haemoglobin, washed in PBS and total genomic DNA was extracted using Qiagen’s QIAamp DNA blood kit as per manufacturer’s instructions.

### Quantitative PCR

DNA controls for *Anaplasma phagocytophilum* and *Babesia divergens* were obtained from the Tick Cell Biobank, University of Liverpool. Dilutions were performed for these samples to determine the limit of detections. qPCR analysis of the serial dilution and tick samples were assessed using the Quantinova probe kit (Qiagen) according to the manufacturer’s instructions [34] with primer.

/probe combinations as described [35, 36] (Supplemental Table S2).

### Conventional PCR

To confirm any qPCR positives, PCR was conducted to test the *Babesia*-genus primers targeting *18S rRNA* and *Bath-F* (Supplemental Table S2) [36, 37]. PCR amplification was carried out in a total volume of 25 µl, containing 1.5 µl of sample DNA, 5 µl of Q5 buffer, 0.5 µl of dNTPs, 16.5 µl of water, 0.25 µl of Q5 High Fidelity DNA Polymerase (New England Biolabs), 0.125 µl of forwards primer and 0.125 µl of reverse primer (100 µM). Amplification was performed with the following conditions: hold cycle of 90 °C for 30 s followed by 35 repeats of 98 °C for 10 s, 54 °C for 30 s and 72 °C for 55 s and held at 72 °C for 2 min, on a Kyratec SC300G-R2 PCR thermocycler. Results were analysed in a 1% agarose gel in Tris–acetate–EDTA (TAE). Gels were stained using SYBR Safe DNA Gel Stain (Thermofisher), while Hyperladder 1 kb and Hyperladder 100 bp (Bioline) were used for size determination. Gels were routinely run at 120 V for 30 min and visualised using a Gel Doc XR + Gel Documentation System (BIO-RAD) and Imagelab.

### RPA primer design

Sequences of *Babesia divergens HSP70* (EU185809.1), *cox1* (MG344907.1), and *18S rRNA* (LC477139.1) genes available from Genbank NCBI were aligned using the alignment editor Aliview (Windows version 1.28) and potential primers generated via Primer3. Primers (Supplemental Table 2) were based on the parameters for optimal RPA primers in the TwistAMP design manual [38]: a GC content between 70% and 30%, a primer T_m_ between 50 and 100 °C and no more than 5 mononucleotide repeats, and were synthesised by IDT (Integrated DNA Technologies). Primer stocks were diluted to 100 µM with Milli-Q water and further diluted to 10 µM prior to their use.

### Primer screening

Primer screening (Supplemental Table S3) using RPA Basic liquid kit (TwistDx) was performed according to the manufacturer’s instruction [39] with the single modification that 2.5 µl of each primer was added to the primer mix, and the reaction was run uninterrupted at 39 °C for 30 min in a heat block. To enable visualisation of amplified product with gel electrophoresis, an additional cycle of 20 min at 99 °C was conducted to denature the proteins and halt the reaction.

### Probe design

Lateral flow (LF) probes were designed to sit between the forward and reverse primers without overlapping. The probe was designed as advised by TwistDx [38], where a sequence of 46–52 nucleotides long was chosen, and a tetrahydrofuran (THF) residue was inserted at least 30 bp from the 5’ end and 15 bp from the 3’ end. The 5′ end of the probe was labelled with 5-carboxyfluorescein (FAM), while a C3-spacer was added at the 3′ end. In addition, the primer on the strand opposite the probe was modified with a biotin label at the 5′ end. The sequence and primers were imported into the online Multiple Primer Analyser [40] and sequences were repeatedly tested for self-dimerisation and cross-dimerisation. Potential probe sequences were also chosen to lie between as many primer pairs as possible to maximise success, and were analysed via BLAST search for any cross reactions over 95% identity. These sequences (Supplementary Table S4) were synthesised by IDT.

### RPA-LF

Most RPA-LF reactions were conducted via modifications to RPA Basic Solid and RPA Basic Liquid protocols. When using the TwistDx RPA Basic solid kit, using 100 µM stocks, a primer–probe master mix consisting 4 µl of forward and biotin primer each, 2 µl of probe and 90 µl of nuclease-free water was made per sample and stored at − 20 °C. For a 10 × RPA Enzyme master mix, 295 µl of TwistDx 2 × reaction buffer was added to 30 µl of endonuclease IV (New England Biolabs). A total of 40.5 µl of enzyme master mix was added to each TwistAMP Basic lyophilised pellet tube alongside 5 µl of primer–probe master mix and 2 µl of the DNA sample (~ 2 to 10 ng/ul). When using the TwistDx RPA liquid kit, 100 µM primers and probe stocks were diluted to 10 µM. Per reaction, 21.5 µl of master mix containing 2.4 µl of water, 3 µl of Endonuclease IV, 0.6 µl of diluted probe, 3 µl of dNTPs, 2.5 µl of core reaction mixture, 5 µl of E-MIX, 2.5 µl of diluted forward primer and 2.5 µl of diluted biotinylated reverse primer was added to 1 µl of sample DNA. Using either kit, the reaction is initiated by adding 2.5 µl of magnesium acetate and running at 39 °C for 45 min. After incubation, a PCRD Lateral flow cassette and PCRD FLEX LF dipstick (Abingdon Health, UK) were used to detect RPA products in accordance with the manufacturer's instructions [41]. These tests have 2 test lines and 1 control line and will detect amplicons that are labelled with DIG/Biotin and/or FITC (or FAM)/Biotin.

### Sensitivity and specificity

To evaluate the sensitivity of the RPA Assay, 2 µl of *Babesia divergens* control DNA was added to 18 µl of nuclease-free water in 10 × serial dilutions, resulting in concentrations between 10^1^ and 10^–7^ ng/µl. Probe specificity was investigated experimentally using genomic DNA extracted from samples of ticks *Dermacentor reticulatus, Ixodes ricinus,* and from in vitro cultures of laboratory strains of *Plasmodium falciparum* (3D7 laboratory strain), *Plasmodium knowlesi* (A1.H1), and *B. divergens* (Rouen 1987).

## Results

### Primer testing and probe selection for *B. divergens* detection

**Fig. 1:**
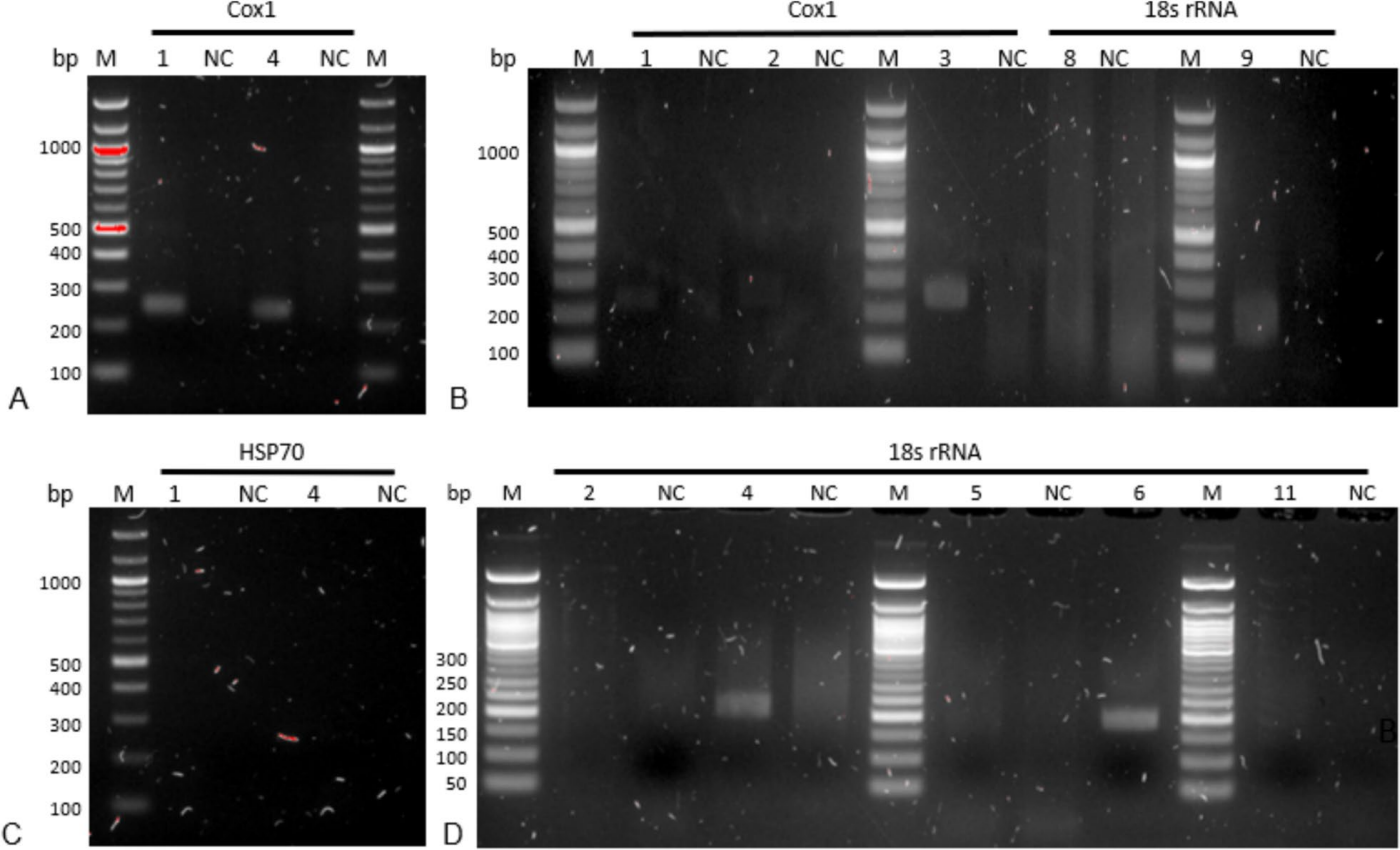
Agarose gel electrophoresis screening of RPA primers for *B. divergens* detection. **A** *cox1* primer sets (primer information in Supplementary Table S3) showing successful amplification of expected amplicon sizes: COX1-1 (233 bp) and COX1-4 (249 bp). **B** Successful amplification of COX1-3 (246 bp) with faint amplification of 18S rRNA-9 (174 bp). **C** Failure of HSP70 primers 1 and 4 to amplify target sequences. **D** *18S rRNA* primer evaluation with successful amplification by primer sets 4 (216 bp) and 6 (195 bp). Target genes are labelled above each gel panel with primer pair numbers indicated above individual wells. *M* molecular weight marker (sizes indicated in bp), *NC* no-template control. Ran on *B. divergens* DNA

Thirteen pairs of RPA primers targeting Heat Shock Protein 70 (*hsp70*), mitochondrial cytochrome-c oxidase subunit 1 gene (*cox1*), and ribosomal 18S rRNA (*18S rRNA*) were tested to identify the optimal primer sets for *B. divergens* detection through RPA assays (Supplementary Table S3). The primers targeting *hsp70* failed to produce the expected banding pattern in the presence of the positive control and were, therefore, unsuitable for further use (Fig. 1C). For the *cox1* target, primer sets Cox1-1, Cox1-3, and Cox1-4 successfully produced bands of the predicted sizes (233, 246 and 249 bp, respectively) (Fig. 1A, B). Since Cox1-1 and Cox1-3 primers amplified overlapping regions, a probe was designed that could be used with both sets (Supplementary Table S4). Among the *18S rRNA* primer pairs, sets 4, 6, and 9 generated the expected bands of 216, 195 and 174 bp, respectively (Fig. 1B, D). Probes were designed for 18S rRNA-4 (based on Genbank number EF458223.1) and 18S rRNA-6 (based on Genbank number LC477139.1) (Supplementary Table S4). Before testing the *cox1* and *18S rRNA* probes, BLASTn searches were conducted to check for potential cross reactions with other species in silico (Table S4). Cox1-4 assays showed significant cross-reactivity with species both within and outside the *Babesia* genus, whereas Cox1-1 and Cox1-3 demonstrated minimal cross-reactivity, only reacting with *B. capreoli* (MG344976.1), due to the high sequence similarity between both species (99.83%). Similarly, the probe and primer set for 18S rRNA-6 were preferred over 18S rRNA-4, as set 4 shared over 95% identity with several species outside the *Babesia* genus (Table S4).

### Comparing the performance of the RPA-LF cassette and the RPA-LF FLEX dipstick

**Fig. 2:**
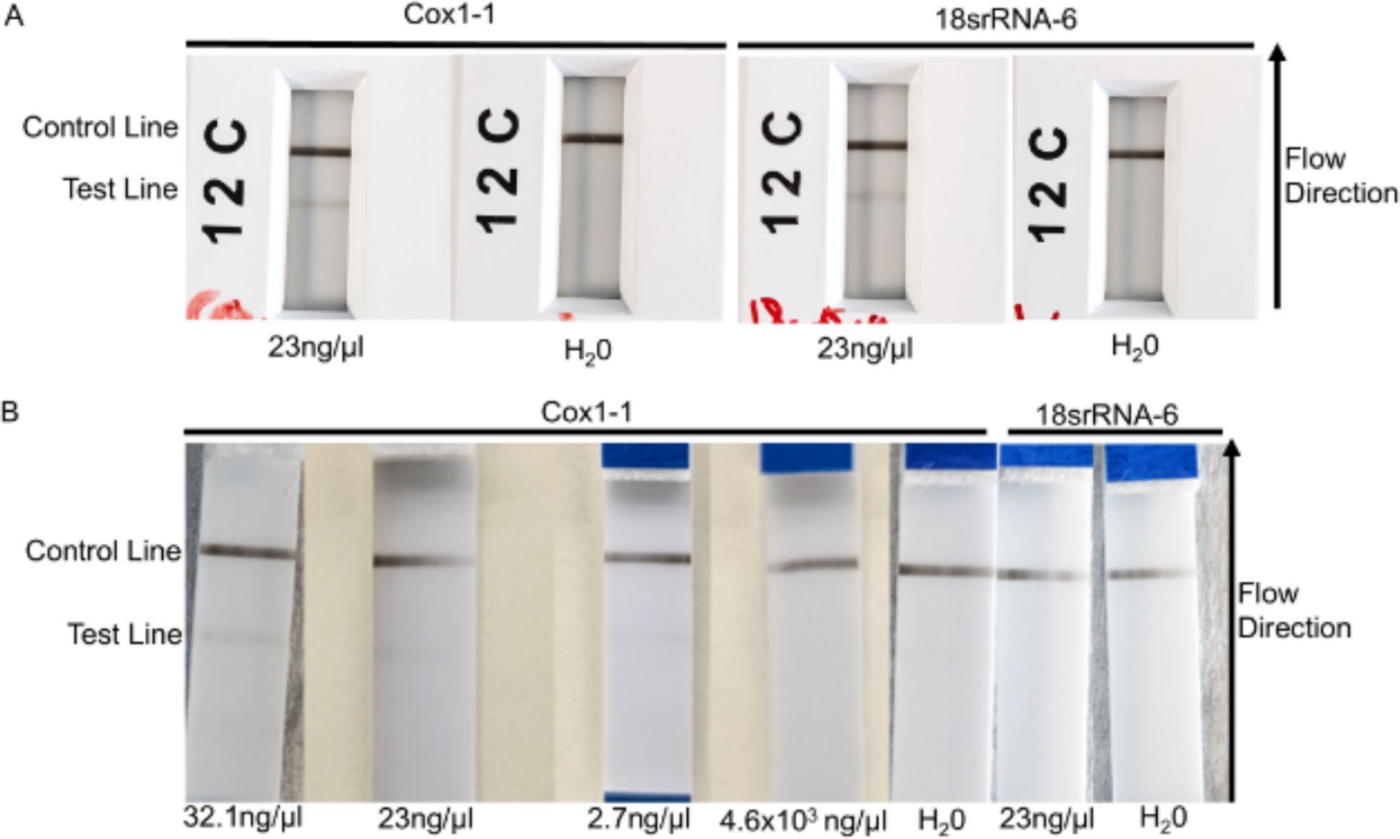
Comparative performance of RPA-LF detection platforms. RPA products detected using COX1–Probe 1 and 18S rRNA–Probe 6 (primer/probe information in Supplementary Table S4) with **A** standard PCRD lateral flow cassette and **B** PCRD FLEX dipstick. Positive results show visible test lines; negative controls show only control lines. H₂O = no-template control. Concentrations shown in ng/μL

To compare the performance of both probes, RPA reactions using Probes 1 and 6 were tested on both the standard PCRD LF cassette and the PCRD FLEX dipstick against the *B. divergens* positive control (2.7 ng/µl) and the water negative control. The PCRD FLEX dipsticks was tested as it could serve as a more cost-effective and field-portable alternative to the cassette for subsequent lateral flow visualisation. Using the PCRD LF cassette, reactions with both Probe 1 and Probe 6 were able to successfully detect RPA amplification, and a positive line was observed (Fig. 2A). Conversely, the PCRD dipstick was unable to detect amplification with Probe 6 in the presence of the positive control and produced a faint positive line for Probe 1 (Fig. 2B). Due to the limited sensitivity observed with the PCRD FLEX dipstick format, further comparative analysis was discontinued. All subsequent experiments were conducted using the standard PCRD LF cassette, which, although slightly more expensive per test than the dipstick format (£2.80 vs £2.00), offers greater robustness, easier result interpretation, and a reduced risk of user error, advantages that are particularly important for reliable field deployment.

### RPA-LF sensitivity and specificity

**Fig. 3:**
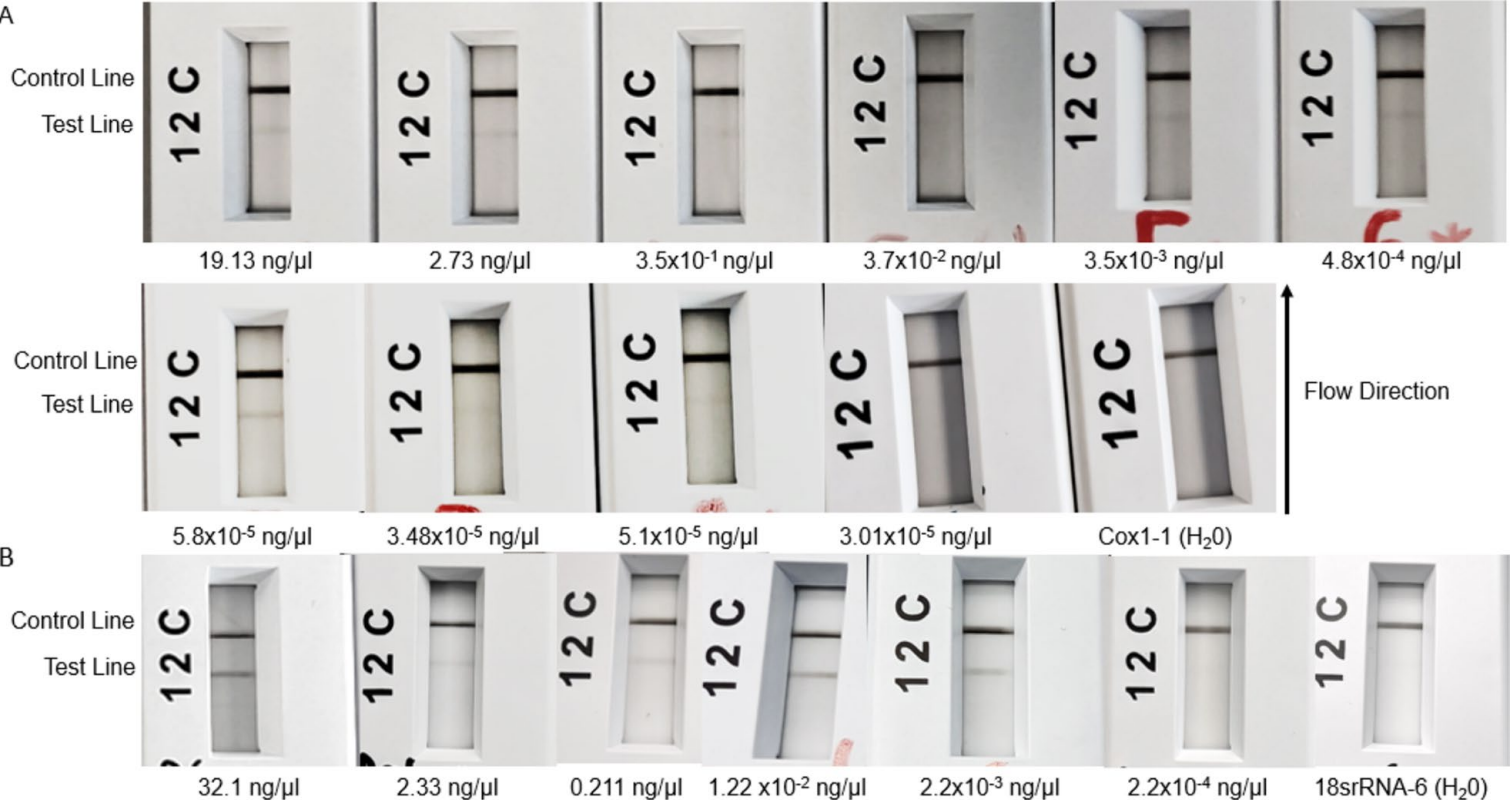
Limit of detection determination for *B. divergens* RPA-LF assays. Serial tenfold dilutions of *B. divergens* genomic DNA tested in duplicate using **A** COX1–Probe 1 and **B** 18S rRNA–Probe 6. Concentrations shown in ng/μL. H₂O = negative control

To assess the sensitivity of the assay, RPA-LF tests targeting the *cox1* and *18S rRNA* genes were performed using tenfold serial dilutions of *B. divergens* DNA. Each concentration was tested in duplicate. In the *cox1* assay, the limit of detection (LOD) was determined to be 5.8 × 10⁻^5^ ng/µL, as positive lines remained faintly visible at this concentration but failed to produce a result at 5.11 × 10⁻^5^ ng/µL (Fig. 3A). For the *18S rRNA* assay, initial tests showed visible positive lines down to 2.2 × 10⁻^3^ ng/µL (Fig. 3B). However, across replicates, consistent and reproducible bands were only observed down to 1.22 × 10⁻^2^ ng/µL. Below this concentration, positive lines were usual faint or absent. This difference in observed LOD can be attributed to possible variations due to individual test cassettes or other experimental variables, including primer binding strength, amplification efficiency and copy number.

**Fig. 4:**
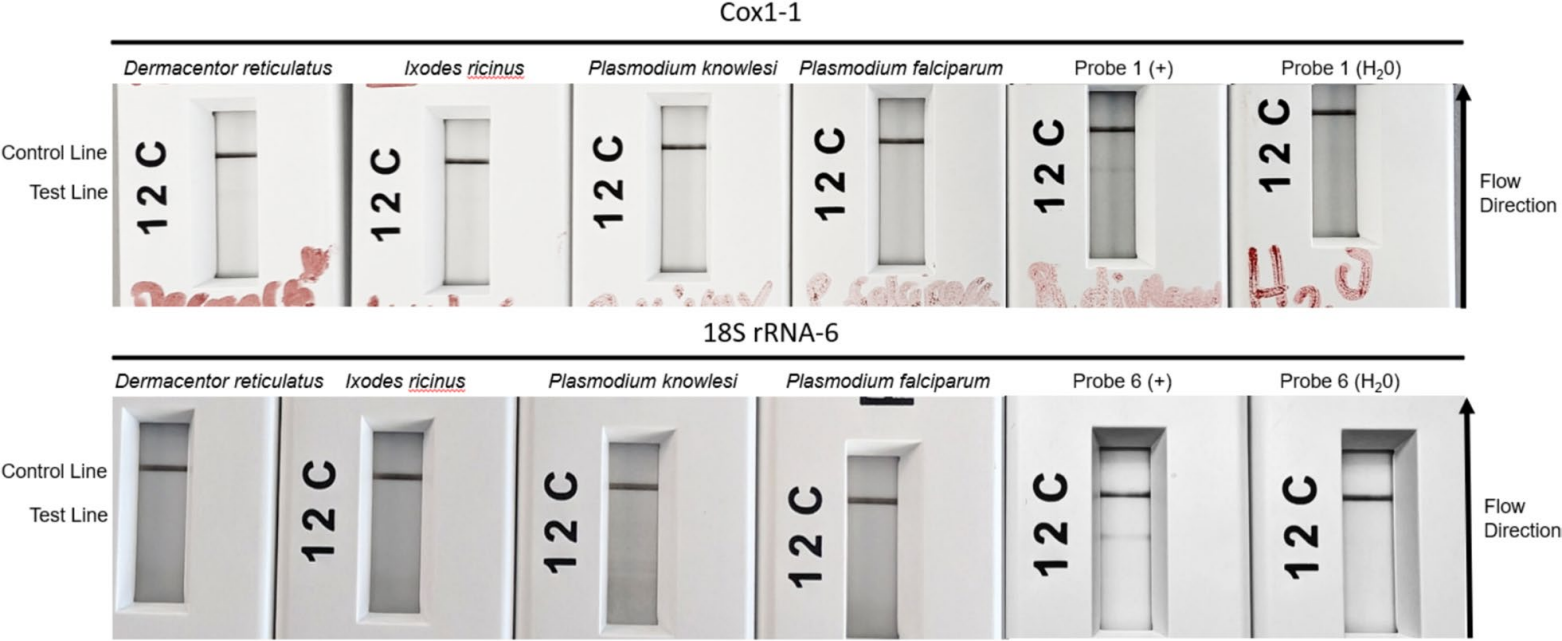
Specificity evaluation of *B. divergens* RPA-LF assays. Cross-reactivity testing using **A** COX1–Probe 1 and **B** 18S rRNA–Probe 6 against genomic DNA from potential cross-reacting species. Both assays showed specific detection of *B. divergens* without cross-reaction to tick species (*D. reticulatus, I. ricinus*) or related apicomplexan parasites (*P. falciparum, P. knowlesi*). (+) = *B. divergens* positive control; H₂O = negative control

To evaluate the specificity of the *cox1* and *18 s rRNA* RPA-LF assays, RPA amplification was performed using purified genomic DNA from various hosts and related pathogens. In both tests (Fig. 4), there was no cross-reaction with DNA from *Dermacentor reticulatus*, *Ixodes ricinus*, *Plasmodium falciparum* and *Plasmodium knowlesi*, with only the genomic DNA of *Babesia divergens* displaying a positive result. Although DNA from other *Babesia* species was not available for testing, these results demonstrate that the RPA-LF assays specifically detect *B. divergens* without cross-reacting with other related apicomplexan parasites or tick host species.

### Detection of Babesia divergens in field samples

**Fig. 5:**
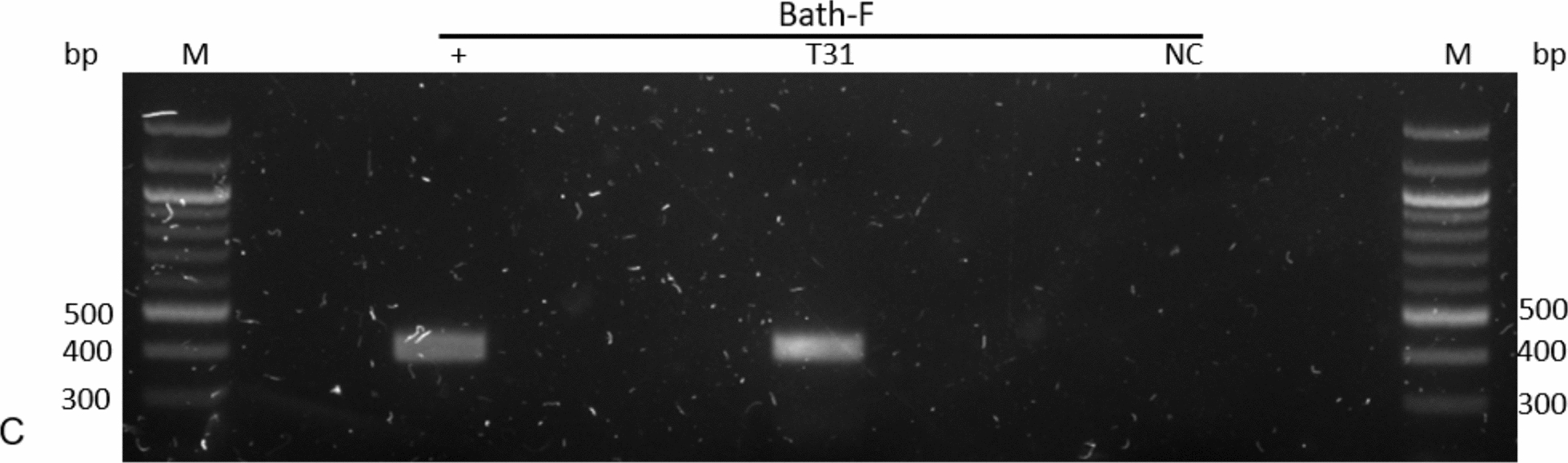
PCR confirmation of qPCR-positive sample (T31). Gel electrophoresis showing amplification products using BATH-F *Babesia*-genus primers (expected size: ~ 400 bp). Primer targets are labelled above each gel panel M = molecular weight marker; (+) = positive control; NC = negative control; T31 = qPCR-positive field sample

A total of 249 *Ixodes ricinus* nymphs were collected from the field and organised into 78 pools of three nymphs each for qPCR analysis. A single three-nymph pool tested positive for *B. divergens* (Cycle threshold 35, equating 3.02 × 10^–5^ ng/µl). Due to the pooled sampling approach, the exact number of infected ticks could not be determined. Based on this result, the estimated nymphal infection prevalence (NIP) ranges from 0.4% to 1.2%. The qPCR-positive pool was subsequently confirmed using conventional PCR (Fig. 5) and Sanger sequencing of PCR product to confirm *Babesia*-genus DNA content. BLAST analysis (NCBI) of the Sanger sequence revealed > 96% identity with both *Babesia divergens* and *Babesia capreoli*; when four ambiguous bases were excluded, this corresponded to 100% identity, indicating a high degree of similarity between these species [42]. Comparative analysis with *Babesia* sequences reported from UK ticks (*n* = 15) [10] showed that, after removal of missing bases, the sequence was identical to one *B. divergens* sequence, differed by 2 SNPs from four *B. capreoli* sequences, by 13 SNPs from nine *B. venatorum* sequences, and by 15 SNPs from *B. odocoilei*. While these results are consistent with *B. divergens*, the high degree of sequence similarity between *B. divergens* and *B. capreoli* means that the positive sample cannot be definitively assigned to either species without additional molecular markers, consistent with previous UK surveillance studies [10, 26, 42]. All samples tested were negative for *Anaplasma phagocytophilum*.

**Fig. 6:**
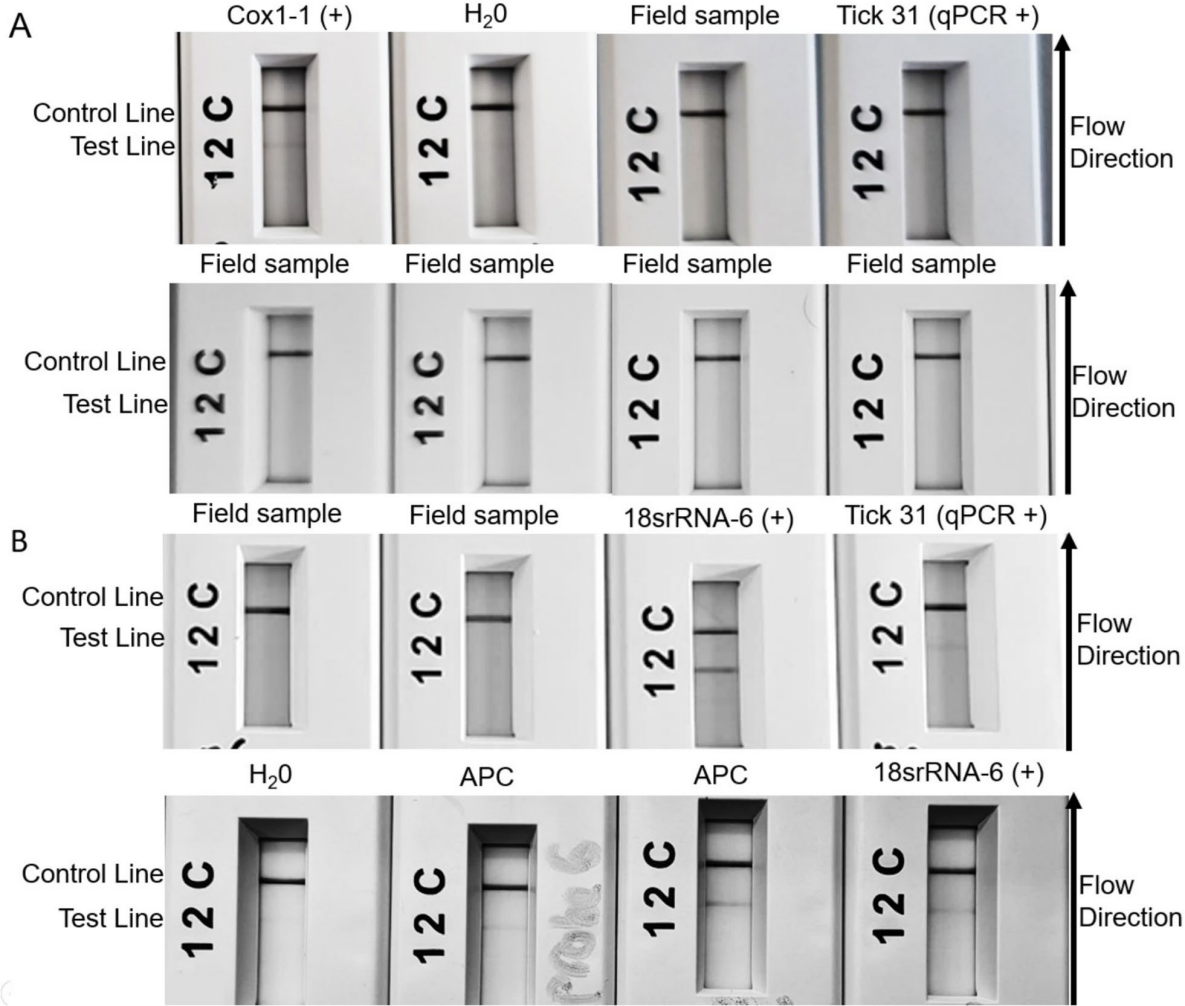
RPA-LF validation using field-collected tick samples. Performance comparison of **A** COX1–Probe 1 and **B** 18S rRNA–Probe 6 assays. Sample origins (pools): Richmond Park, Bushy Park. The *18S rRNA* assay successfully detected the qPCR-positive sample T31 (pool) twice, while *cox1* assay failed to detect this low-concentration sample. *APC* artificially spiked positive control; (+) = positive controls; H₂O = negative control

To assess the performance of RPA-LF assays in field-collected samples, both the Cox1-1 and 18S rRNA-6 assays were used to evaluate ten pools, including the one positive by qPCR. All pools that tested negative by qPCR were correctly identified as negative by both developed RPA assays. However, the Cox1-1 assay failed to detect *Babesia* DNA using the qPCR-positive pool, resulting in a false negative (Fig. 6A). In contrast, the 18S rRNA-6 RPA assay detected this sample as positive, albeit with variable band intensity between replicates. The 18S rRNA-6 assay also successfully identified *Babesia* DNA in two artificially spiked positive controls (Fig. 6B), making the newly developed *18S rRNA* RPA-LF the most reliable field-adaptable diagnostic tool for detecting active *B.divergens* infections from DNA isolated from whole vector sampling.

## Discussion

*Babesia divergens* is of significant zoonotic importance, with infections documented in both deer and cattle across Europe, and occasional severe cases in human infections [13]. Human cases, while rare (> 60 cases in Europe) [10], include recent infections in Scotland [43] and Southwest England in 2020 [13], demonstrates an ongoing transmission risk despite a low prevalence of *Babesia* spp. in UK *Ixodes ricinus* populations [9, 10, 26], underscoring the growing need for effective field-adaptable surveillance tools. This study thus developed and validated a novel RPA-LF molecular diagnostic tool that could enhance current surveillance capabilities with field testing on collected ticks to demonstrate its practical relevance.

In this study, two RPA-LF assays targeting *cox1* and *18S rRNA* were designed for the rapid and sensitive detection of *Babesia divergens,* while *hsp70* primers failed to amplify the target using *B. divergens* DNA and were discontinued. Serial dilution showed that the *cox1* assay demonstrated a 100-fold greater sensitivity than that of the *18 s rRNA* assay with limits of detection of 10^−5^ ng/µl and 10^−3^ ng/µl, respectively. As sensitivity was determined using serial dilutions of genomic DNA, it is expressed in terms of total DNA concentration and, therefore, represents an approximate detection threshold rather than an absolute genome copy number. Accordingly, it is most appropriately compared with studies reporting mass-based sensitivity estimates. Although not as sensitive as previously reported RPA-LFs for *B. vogeli* RPA-LF (LOD: 1 × 10⁻⁷ ng/µl)[21], the sensitivity achieved was in line with assays for *Anaplasma phagocytophilum* (1.77 × 10⁻^5^ ng/µl), *Plasmodium falciparum*, and *Puccinia striiformis* (both 10⁻^5^ ng/µl) [44–46].

Specificity testing confirmed that both *cox1* and *18S rRNA* RPA-LFs accurately detected *B. divergens* without cross-reactivity with other apicomplexan DNA (*Plasmodium falciparum*, *P. knowlesi*) or with local tick species DNA (*Ixodes ricinus*, *Dermacentor reticulatus*). This aligns with prior *Babesia* RPA-LF studies showing no cross-reaction with other apicomplexan or tick vectors DNA [22–24]. However, further validation is needed testing other *Babesia* spp. (*B. gibsoni*, *B. microti*, *B. bovis*, *B. orientalis*, *B. bigemina*) DNA samples and related piroplasms such as *Theileria* species present in the UK [21–24]. As most emerging tick-borne pathogens are bacterial or viral and, therefore, lack *18S rRNA* regions [8], cross-reactivity is unlikely.

The RPA-LF assay for both *cox1* and *18S rRNA* were validated on ten field samples, including the qPCR-positive pool estimated to a concentration of 10^−5^ ng/µl. The *18S rRNA* probe was able to successfully detect this sample alongside two further spiked samples demonstrating diagnostic validity with neither false positives nor false negatives. In contrast, the *cox1* probe was unable to detect the field positive sample indicating a lower sensitivity that requires further optimisation. This result may reflect several factors. First, the positive sample may contain genetic variation that could have affected detection by the cox1 assay due to sequence variation in primer or probe-binding regions reducing amplification efficiency. In silico BLAST analysis identified a few *Babesia divergens* and *Babesia capreoli* sequences with a few single SNPs within the cox1 probe-binding region, which could decrease binding affinity, while the *18S rRNA* primer/ probe sequences were conserved. Although RPA is generally tolerant to sequence mismatches, such variation may still impair performance under low-template conditions. In addition, stochastic effects near the limit of detection, as well as the presence of inhibitors in tick-derived extracts, may further influence assay sensitivity. These combined factors may explain the comparatively better performance of the 18S rRNA assay in field samples, despite its potentially lower analytical sensitivity under controlled conditions.

The success of the *18 s rRNA* assay in the detection of field and spiked samples suggests a sensitivity at approximately 3.02 × 10⁻^5^ ng/μL. The results aligns with scientific literature, with the RPA-LF of *Babesia microti*, *B. vogeli*, *B. gibsoni* and *B. orientalis* yielding equal or superior sensitivity compared to conventional PCR [21–24]. Further improvement should focus on *cox1* primer and probe refinement.

The *18S rRNA* RPA-LF assays demonstrated fast and reliable detection of *B. divergens*, completing within 45 min at 39 °C using pre-prepared reagent mixes. This represents a substantial advance over PCR and LAMP, which require thermal cyclers or constant heat sources. Its portability and rapid turnaround make it particularly valuable for xenomonitoring in field settings and remote, resource-limited areas, where conventional laboratory infrastructure is lacking. However, in this study, we used a column-based DNA extraction method, which limits true point-of-sampling deployment.

A key barrier to field deployment remains the DNA extraction process, as current protocols rely on commercial kits requiring laboratory equipment, such as [33] vortexers and centrifuges, which limit field applicability. However, RPA’s resilience to crude DNA extracts and biological inhibitors that typically compromise PCR performance [16] offers significant advantages for field deployment capable of operating with nucleic acids from blood, serum, faecal, nasal, and vaginal swabs, plasma, food, plants, animal tissue, milk, stool and urine [16]. This robustness also makes it compatible with less refined sample preparations, such as in the detection of malaria parasites from *Anopheles* mosquitoes extracted using a crude DNA extraction protocol [18]. Adapting similar extraction protocols for tick samples could streamline field diagnostics and allow for more autonomous surveillance efforts. This compatibility with minimally processed blood samples, such as EDTA or heparinised cow blood samples taken in situ and stored refridgerated in the presence of anticoagulants, would lay the groundwork for a future point-of-care, clinical or veterinary diagnostic tool to complement the limitations of existing serology and microscopy-based diagnostics [11, 12].

Finally, tick pooling complicates prevalence estimates. Since ticks were grouped in threes, a single positive pool could reflect one to three infected ticks, implying a nymphal infection prevalence between 0.4% and 1.2%. This range is consistent with previous reports in southern England (0.3–0.46%) and falls within European estimates (0–2.3%) [10, 26]. Larger-scale studies are needed to more robustly assess assays performance and to accurately determine the true prevalence of *Babesia divergens* and *Babesia divergens-*like in the study areas. In addition, evaluation under a wider range of field conditions will be important to fully validate surveillance utility. For example, sampling in cattle-grazing areas may provide a more targeted approach for detecting *B. divergens*. While this species is classically associated with cattle, it has also been detected in ticks from deer-associated environments in Europe, including the UK, and there is some evidence of its presence in deer, although their role as competent reservoirs remains unclear. The inclusion of larval stages in future studies will also be important to improve understanding of transmission dynamics and early-stage infection prevalence. Furthermore, investigating deer-associated environments, alongside distinguishing *B. divergens* and *Babesia capreoli*, will be important to clarify their respective ecological roles and understand the real risk of *B. divergens* for public health risk. The successful use of RPA-LF in detecting *Babesia divergens*-like parasites in a pooled nymph sample highlights the capability of RPA-LF for monitoring low-prevalence pathogens in field conditions and its its potential for future implementation in routine vector surveillance and early pathogen detection.

## Conclusions

This study successfully developed a RPA-LF assay for the detection of *Babesia divergens-*like parasites, providing a simple, rapid, point-of-sampling tool without the equipment and expertise constraints of PCR. When combined with simplified or crude DNA extraction approaches, the assay has strong potential for true point-of-sampling deployment. By producing easily interpretable bands in under 45 min at 39 °C, this project represents a significant step towards a portable molecular diagnostic tool capable of supporting xenomonintoring efforts in diverse surveillance settings. Field validation demonstrated the assay's practical utility by detecting *Babesia divergens*/*B. capreoli* in a tick sample from an urban green space, eventually providing future researchers and public health practitioners with a valuable tool for rapid pathogen screening in resource-limited or remote settings, where traditional laboratory infrastructure is unavailable.

## Supplementary Information

Additional file 1.

## Data Availability

All data generated or analysed in this study are included in this published article and supplementary information file.
