# Supplementary material for: A recombinase polymerase amplification lateral flow assay (RPA-LF) for detection of Babesia divergens-like parasites
Source: Parasit Vectors. 2026 Jul 27;19:338. doi: 10.1186/s13071-026-07549-0 (PMC13479736; doi:10.1186/s13071-026-07549-0)
Supplement: Supplementary file 1 [file 13071_2026_7549_MOESM1_ESM.docx]

**Supplemental information**

**Supplementary Table S1.** Sampling site coordinates for tick collection in Bushy Park and Richmond Park, Greater London, including GPS latitude and longitude for all locations sampled.

| Park | Location | Coordinates (Longitude, Latitude) | Date and time visited |
| --- | --- | --- | --- |
| Richmond Park A | 1) Sawers hill entrance | 51.449, -0.282 | 3/6/24 1700 |
|  | 2) Saw pit plantation | 51.448, -0.279 | 3/6/24 1830 |
| Bushy Park A | 3) Near TAFC pitch | 51.420, -0.348 | 5/6/24 1700 |
|  | 4) Alongside Cobblers Walk | 51.421, -0.348 | 5/6/24 1715 |
|  | 5) Streamside | 51.422, -0.350 | 5/6/24 1726 |
|  | 6) Canal plantation approach | 51.423, -0.350 | 5/6/24 1741 |
| Bushy Park B | 7) Heron pond | 51.413, -0.330 | 16/6/24 1730 |
|  | 8) Leg of mutton Pond | 51.413, -0.328 | 16/6/24 1800 |
| Richmond Park B | 9) Kidney woods | 51.448, -0.293 | 19/6/24 1730 |
|  | 10) Spankers hill | 51.441, -0.265 | 19/6/24 1945 |
|  | 2) Saw pit plantation | 51.448, -0.279 | 19/6/24 2030 |
|  | 1) Sawers hill entrance | 51.449, -0.282 | 19/6/24 2100 |

**Supplementary Table S2.** Primer and probe sequences used for qPCR and conventional PCR, and including forward and reverse primers for targeted *Babesia* and *Anaplasma* gene regions

| Species | Target | Name | Sequence 5' to 3' | Source |
| --- | --- | --- | --- | --- |
| cPCR | | | | |
| *Babesia* genus | 18s rRNA | BabGF2 | GYYTTGTAATTGGAATGATGG | [36] |
|  |  | BabGR2 | CCAAAGACTTTGATTTCTCTC |  |
|  |  | Bath-F | TAAGAATTTCACCTCTGACAGTTA | [37] |
|  |  | Bath-R | ACACAGGGAGGTAGTGACAAG |  |
| qPCR | | | | |
| *Babesia divergens* | *hsp70* | Bab_di_hsp70_F | CTCATTGGTGACGCCGCTA | [36] |
|  |  | Bab_di_hsp70_R | CTCCTCCCGATAAGCCTCTT |  |
|  |  | Bab_di_hsp70_P | AGAACCAGGAGGCCCGTAACCCAGA |  |
| *Anaplasma phagocytophilum* | *msp2* | An_ph_msp2_F | ATGGAAGGTAGTGTTGGTTATGGTATT | [35] |
|  |  | An_ph_msp2_R | TTGGTCTTGAAGCGCTCGTA |  |
|  |  | An_ph_msp2_p | TGGTGCCAGGGTTGAGCTTGAGATTG |  |

**Supplementary Table S3.** Results of RPA primer screening of *hsp70, cox1* and *18S rRNA* target regions, indicating amplification success or failure for each primer pair.

| **Target** | **Name** | **Sequence 5’-3’** | **Comments** |
| --- | --- | --- | --- |
| *18s rRNA* | Babdiv_18srRNA_F2 | ACTAATGTCGAGATTGCACTTCG | Band produced |
|  | Babdiv_18srRNA_R2 | CCCCAACCGTTCCTATTAACC |  |
|  | Babdiv_18srRNA_F4 | TTTGCGTGGTGTTAATATTGACT | Band Produced |
|  | Babdiv_18srRNA_R4 | TGACAGTTAAATACGAATGCCCC |  |
|  | Babdiv_18srRNA_F5 | ACTAATGTCGAGATTGCACTTCG | No bands |
|  | Babdiv_18srRNA_R5 | CCCCAACCGTTCCTATTAACC |  |
|  | Babdiv_18srRNA_F6 | TTTGCGTGGTGTTAATATTGACT | Band Produced |
|  | Babdiv_18srRNA_R6 | CCCAACCGTTCCTATTAACCATT |  |
|  | Babdiv_18srRNA_F8 | CAGAGTAACAATTGGAGGGCA | Contaminated smear |
|  | Babdiv_18srRNA_R8 | AGTCAATATTAACACCACGCAAA |  |
|  | Babdiv_18srRNA_F9 | GGGGCATTCGTATTTAACTGTCA | Band Produced |
|  | Babdiv_18srRNA_R9 | CTCCAATCCCTAGTCGGCAT |  |
|  | Babdiv_18srRNA_F11 | ACTAATGTCGAGATTGCACTTCG | No bands |
|  | Babdiv_18srRNA_R11 | CCCCAACCGTTCCTATTAACC |  |
|  | Babdiv_18srRNA_R13 | GACCTCCAATCCCTAGTCGG |  |
| *hsp70* | Babdiv_hsp70_F1 | CTTCACTGACACTGAGCGTC | No bands |
|  | Babdiv_hsp70_R1 | CCAACACCGTTGATGACCTT |  |
|  | Babdiv_hsp70_R3 | GGTTACTTCGATGGTGGGCT |  |
|  | Babdiv_hsp70_F4 | CAAGAGGCTTATCGGGAGGA | No bands |
|  | Babdiv_hsp70_R4 | GGATGAAATTTCCTCGGGGT |  |
| *cox1* | Babdiv_COX1_F1 | GCTTTTGGTCTTGTGGTTGC | Produced Band |
|  | Babdiv_COX1_R1 | GACCACACTGTAGGAATCAACC |  |
|  | Babdiv_COX1_F2 | TTGGTCTTGTGGTTGCTTGT | Produced Faint Band |
|  | Babdiv_COX1_R2 | GCGGTTAAAACAATTGACCACA |  |
|  | Babdiv_COX1_F3 | TGTGTTGAATTGGGGAGTGG | Produced Strong Band |
|  | Babdiv_COX1_R3 | CGGCAATGACAAAAGTAGCAAA |  |
|  | Babdiv_COX1_F4 | AGGATGTCTAGTATGGGCACA | Produced Band |
|  | Babdiv_COX1_R4 | ACATCAACACCAGCATTTCCA |  |

**Supplementary Table S4.** RPA-LF probe sequences, primer-probe combinations, and BLASTn identity matches for *cox1* and *18S rRNA* assays. Abbreviations: FAM- 6-carboxyfluorescein, Bio- Biotin modification, THF- abasic Tetrahydrofuran residue, SpC3-3’C3 spacer.

| Name | Sequence 5’ to 3’ | BLAST SEARCH  and Accession Numbers |
| --- | --- | --- |
| Babdiv_COX1-PROBE1 | /56-FAM/TGGGGAGTGGAACTGGGTGGACATTGTACC/THF/TCCTTTATCTACTTCTA/3SpC3/ | *Babesia divergens*MG344956.1 |
| Babdiv_COX1-F1 | GCTTTTGGTCTTGTGGTTGC | *Babesia divergens*MG344979.1  *Babesia capreoli*MG344976.1 |
| Babdiv_COX1-R1 | /5Biosg/GACCACACTGTAGGAATCAACC | *Babesia divergens*MG344979.1 |
| Babdiv COX1-PROBE4 | CACTTCTGGATTAGAAGCAGATACTAGAGCTTTTTTTACAACAACTAC | *Babesia divergens* MG344979.1  *Babesia gibsoni* KP666169.1 |
| Babdiv COX1-F4 | AGGATGTCTAGTATGGGCACA | *Babesia divergens*MG344979.1, *Leucocytozoon pterotenuis* KM610046.1 |
| Babdiv COX1-R4 | ACATCAACACCAGCATTTCCA | *Babesia divergens*MG344979.1  *Babesia capreoli*MG344965.1  *Babesia motasi* KT224420.1  *Babesia bigemina* PP620594.1  *Babesia ovata* LC684773.1  *Babesia hermsii* CP005685.1 |
| Babdiv 18srRNA PROBE4 | AGTAGGACTTTGGTTCTATTTTGTTGGTTTGTGACCTTAATATGGTTAAT | *Babesia divergens* EF458223.1 |
| Babdiv 18srRNA F4 | TTT GCG TGG TGT TAA TAT TGA CT | *Babesia divergens* MT550684.1  *Babesia capreoli* MT151374.1 |
| Babdiv 18srRNA R4 | TGA CAG TTA AAT ACG AAT GCC CC | *Eimeria tenella* PQ142694.1  *Babesia vogeli* PQ136456.1  *Theileria bicornis* LC817764.1  *Sarcocystis spp* PQ120683.1  *Babesia canis* PQ123220.1  *Babesia gibsoni* PQ114142.1  *Babesia divergens* PP759299.1  *Cytauxzoon brasiliensis* PP583821.1  *Babesia caballi* PQ056500.1  *Theileria annulata* PQ056498.1  *Theileria luwenshuni* PQ001989.1 |
| Babdiv 18srRNA PROBE 6 | /56-FAM/TTTGGGATTTATCCCTTTTTACTTTGAGAA/THF/ATTAGAGTGTTTCAAGC/3SpC3/ | *Babesia venatorum* KY213884.1  *Babesia odocoilei* KT279886.1  *Babesia divergens*OL504563.1 |
| Babdiv 18srRNA F6 | TTTGCGTGGTGTTAATATTGACT | *Babesia divergens*MT550684.1  *Babesia capreoli* MT151374.1 |
| Babdiv 18srRNA R6 | /5Biosg/CCCAACCGTTCCTATTAACCATT | *Babesia vogeli* PQ136456.1  *Babesia canis* PQ123220.1  *Babesia gibsoni* PQ114142.1  *Babesia caballi* PQ056500.1  *Babesia vulpes* PQ049253.1  *Babesia bigemina* PP944852.1  *Babesia odocoilei* PP512757.1  *Babesia occultans* PP463882.1  *Babesia motasi* LC799510.1  *Babesia ovis* PP259076.1  *Babesia divergens* EU182595 |
